# Variation in plant functional traits explains the substitution distribution and allocation strategy of *Stipa* species across natural grasslands of Ningxia, Northern China

**DOI:** 10.1002/ece3.70164

**Published:** 2024-08-09

**Authors:** Jun Yang, Xiaowei Li, Junlong Yang, Shuang Yu, Hongmei Zhang, Bo Yang

**Affiliations:** ^1^ College of Forestry and Pratacuture Ningxia University Yinchuan China; ^2^ State Key Laboratory Breeding Base of Land Degradation and Ecological Restoration of Northwest China Ningxia University Yinchuan China

**Keywords:** plant economic spectrum, plant functional traits, *Stipa*, substitution distribution

## Abstract

Functional traits reflect plants' adaptability to their environment, and environmental gradients influence their distribution. But few studies have investigated the link between these traits and species substitution patterns or the relevant ecological factors. We measured the aboveground (leaf) and belowground (root) functional traits of *Stipa* species in 17 plots across natural grasslands in Ningxia in Northern China. Redundancy analysis was used to explore the relationships between *Stipa*'s functional traits and its species substitution distribution. Then, on the species substitution gradient, principal component analysis (PCA) was used to verify and quantify the leaf economic spectrum (LES), root economic spectrum (RES), and whole‐plant economic spectrum (WPES), with the relation between these spectra investigated by fitting standardized major axis regressions. The effects of aboveground, belowground, and whole‐plant ecological factors were quantified and ranked by variance decomposition and hierarchical partitioning. Our results showed that functional traits drive the substitution distribution of *Stipa* species, in being variously coupled with its desert, typical, and meadow steppe habitat types. The leaf, root, and whole‐plant economic spectra of *Stipa* species in desert steppe exhibit a “quick investment‐acquisition” strategy. In typical steppe, the leaf and whole‐plant economic spectra of *Stipa* species correspond to a “fast investment‐acquisition” strategy, whereas the root economic spectrum adopts a “slow investment‐acquisition” strategy. On meadow steppe, the leaf, root, and whole‐plant economic spectra of *Stipa* species similarly adopt a “slow investment‐acquisition” strategy. Finally, when considering the environmental factors involved, we find that the substitution distribution of *Stipa* spp. is chiefly a response to shifting soil patterns, these mainly driven by soil total nitrogen and nitrogen/phosphorus ratio. Collectively, these findings provide an important reference for the ecological restoration and reconstruction of grassland ecosystems, to better understand the relationship between plant functional traits and ecological niche attributes, and thus guide the reasonable restoration of grassland vegetation.

## Graphical abstract

It mainly focuses on the following three points: (1) Establish the relationship between functional traits of *Stipa* and species substitution distribution; (2) economic spectra of leaves, roots, and whole plants of *Stipa* on different grassland types and their relationships; and (3) environmental drivers of *Stipa* substitution distribution.

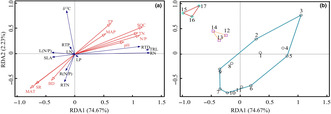

## 1 INTRODUCTION

Functional traits of plants encompass a range of key attributes closely associated with their colonization, survival, growth, and mortality (Reich et al., 2003). Leaf functional traits are inextricably linked to the absorption and utilization of light, water, and nutrients (Wilson et al., 2010). Specific leaf area, leaf nitrogen content, and leaf dry matter content reflect the ability of plants to utilize environmental resources and their conservation of these resources (Toledo et al., 2022). Root functional traits are closely related to soil water and nutrient absorption, physical fixation, resource storage, and nutrient production (Enrique et al., 2023). Furthermore, the nitrogen and phosphorus contents of roots can provide crucial information regarding plants' competitive ability for limiting resources (Williams et al., 2021). The leaf economic spectrum (LES) was introduced by Wright et al. (2004), who borrowed the concept of “investment‐return trade‐off” from economics and applied it to the allocation of resources in plants. Spanning a continuum of plant strategies, “quick investment and return” species are at one end of the LES. These species prioritize rapid returns on investment over structural investment, resulting in a resource acquisition strategy. Conversely, at the other end of the LES are those species that rely on a “slow return on investment” strategy, characterized by high structural investment and delayed returns, which corresponds to a conservative resource strategy. The study of that economic spectrum later expanded to include other plant organs, such as stems and roots, and eventually culminated in the enticing idea of a whole‐plant economic spectrum (WPES) (Reich, 2014). Yet reports on the relation between economic spectra of different plant organs tend to be empirically limited and often inconsistent. For example, Baraloto et al. (2010) detected strong coupling between leaf and stem economic spectra across rainforest tree species. In contrast, Isaac et al. (2017) found that the LES and root economic spectrum (RES) were independent of each other within species of coffee. In particular, although root traits appear to vary with local environmental conditions, leaf traits and their functioning need not adjust accordingly. Reich (2014) argued for a uniform rate of resource acquisition and processing across all plant organs, postulating the existence of an integrated WPES.

The concept of substitution distribution describes an ecological pattern, whereby diverse species engage in competitive replacement across differing habitats. This phenomenon frequently occurs when a species that is predominant in one habitat is progressively displaced by one or more other species (Sterck et al., 2014). Understanding substitution distribution is essential for ensuring ecosystem stability and fostering species diversity. One key factor driving this distribution is the variation in plant functional traits, which enabled species to adapt and survive under different habitat conditions (Pollock et al., 2012). For instance, plants with high drought resistance thrive in arid environments, while those species with efficient photosynthesis and nutrient cycling capabilities perform better in nutrient‐rich soils. The differences in functional traits among species lead to divergence in habitat selection and resource utilization, thereby shaping the species substitution distribution (Maharjan et al., 2021). Although there has been some progress in understanding how functional traits influence plant distribution, such research often focuses on tree species along altitudinal gradients (Heegaard, 2002; Morales‐Saldaña et al., 2021), with grassland plants generally understudied. Chinese grasslands can be categorized into four main types: meadow steppe, typical steppe, desert steppe, and alpine steppe. Ma et al.'s (2011) research indicated that on the Mongolian Plateau, *Caragana sinica* is primarily distributed in meadow steppe and typical steppe, where it is gradually replaced by *Caragana liouana* and *Caragana korshinskii* as one moves southward. Research into substitution distribution among different grassland types often centers on the regularity of replacement, while studies examining the underlying causes of this phenomenon are still relatively rare.

Ningxia temperate grassland has a typical continental semi‐humid and semi‐arid climate and is a recognized key area for ecological construction (Fang & Zhang, 2001). Due to the influence of man‐made destruction and climate change, grassland desertification and degradation have caused economic losses and an ecological environment imbalance (Zhang & He, 2023). *Stipa* species, with their low transpiration rates and robust resilience to adversity, are an ideal choice for ecological reconstruction in arid regions. They effectively utilize water resources, tolerate drought, and barren conditions, a solid foundation for ecological restoration (Heegaard, 2002; Yuan, Li, Han, Huang, et al., 2005; Yuan, Li, Han, Wan, & Zhang, 2005). The diverse species within the *Stipa* genus exhibit variations in their morphology, growth habits, and ecological requirements, allowing them to thrive and propagate under different environmental conditions, thereby increasing species diversity (Bai et al., 2023). Additionally, *Stipa* species are capable of enhancing the physical and chemical properties of soil, as well as its fertility. Through their root systems, these plants increase soil organic matter, improve soil structure and texture, fix nitrogen, and enhance soil fertility (Yuan, Li, Han, Huang, et al., 2005; Yuan, Li, Han, Wan, & Zhang, 2005). In ecologically fragile areas, *Stipa* species help prevent desertification, conserve soil and water resources, maintain ecological balance, and reduce the impact of drought and land degradation (Wang et al., 2024). Therefore, *Stipa* species collectively occupy a critical ecological niche in the Ningxia temperate grasslands, which is crucial for the stability and productivity of those grassland ecosystems. Accordingly, by understanding and utilizing the ecological niche attributes of *Stipa* species, we can effectively employ them for ecological restoration, thereby achieving the sustainable development of grassland ecosystems.

**FIGURE 1:**
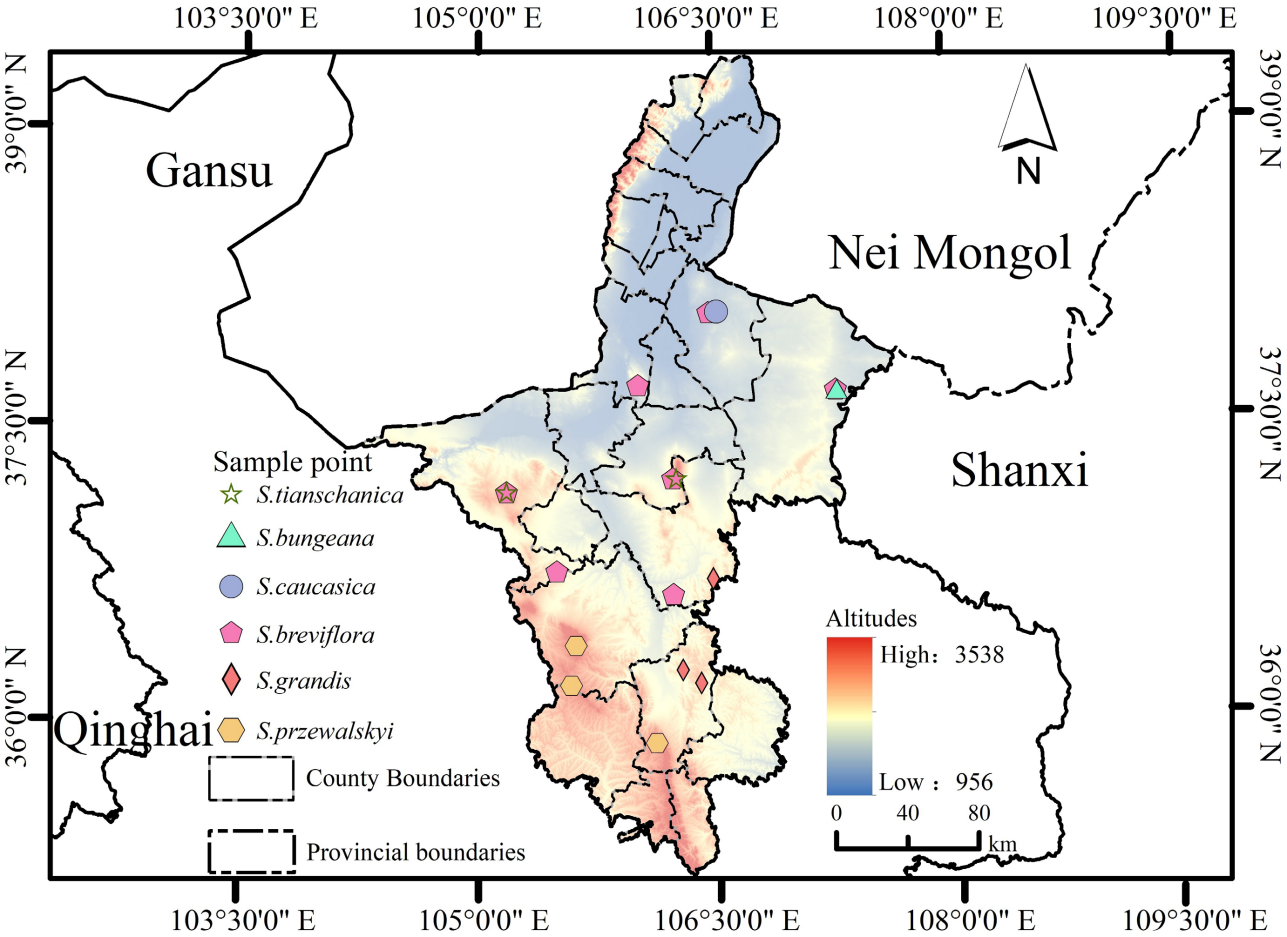
Locations of sampling sites where *Stipa* plant species were sampled in Ningxia temperate grassland, China.

This study aimed to explore how variations in the functional traits of *Stipa* species influence their distribution in Ningxia and to identify the environmental factors shaping that trait variability along geographical gradients. To do that, we established 17 plots across Ningxia, spanning north to south, to sample *Stipa* plants (Figure 1). In doing so, we addressed these questions: (1) How do variations in *Stipa*'s functional traits influence its species distributions across types of grassland in Ningxia? (2) What are the key eco‐economic strategies that *Stipa* species employ to express their leaf, root, and whole‐plant functional traits along the gradient of their substitution distribution within Ningxia's grasslands? (3) What are the dominant ecological factors that drive the alternative distributions of *Stipa* species across the natural grasslands of Ningxia, and how do these factors interact with functional trait variability?

## 2 MATERIALS AND METHODS

### 2.1 Study area

Field survey work was conducted across the extensive Ningxia temperate grassland (35°14′–39°23′ N, 104°17′′–107°39′ E), which is part of the Eurasian Grassland. According to the natural distribution of *Stipa*, a total of 17 natural grasslands with *Stipa* as the dominant species were selected as sampling plots in Ningxia that spanned from south to north (Figure 1). Ningxia, located in the arid and semi‐arid transitional zone, is an area where agriculture and animal husbandry are intertwined, making it sensitive to climate change. Here, the average annual temperature is 4.14~10.72°C, and the annual average precipitation is 198.9 mm, primarily concentrated in July and August; the growing season's annual precipitation ranges from 141 to 249 mm. The average annual sunshine duration is 3004.80 h (Ma et al., 2020), the average annual evaporation is 1928.4 mm, and the water vapor pressure is 3–12 kPa (Yuan et al., 2011).

### 2.2 Field sampling

Each sampling plot, a 10 × 10 m quadrat, was established, and three 1 × 1 m sub‐quadrats were evenly distributed along the diagonal within the quadrat. Inside each 1 × 1 m sub‐quadrat, three healthy and pest‐free dominant *Stipa* plants (Figure 1) were randomly selected. To assess leaf traits, nine complete leaves were randomly chosen from each *Stipa* plant and stored in Ziplock bags containing moist filter paper. Leaf area measurements were conducted within 24 h post‐harvest. Concurrently, roots from the same nine *Stipa* plants within each quadrat were excavated and placed in envelopes for laboratory analysis of root traits. All the *Stipa* plant materials used in the study, including both aboveground and belowground parts, were collected during the peak growing season from June to August. Soil samples were collected using a five‐point sampling method (east, south, west, north, and center) at depths of 0–40 cm for subsequent soil physicochemical property assessments. At the same time, the soil profile was excavated, from which soil samples were collected with the ring knife, and the soil bulk density was determined. All indicators are calculated using the average of the three subquadrate measurements.

### 2.3 Climate data

All climate data were obtained from the World Climate website (http://worldclim.org), whose dataset provides nearly 50‐year dataset of global monthly average weather data at a 1 × 1 km resolution. Following the download of the data for the study area, we calculated the average annual precipitation (MAP), mean annual temperature (MAT), and solar radiation (SR).

### 2.4 Plant functional traits and their measurement

#### 2.4.1 Aboveground *Stipa* traits

The specific leaf area (SLA) is the ratio of a leaf's surface area to its dry weight. Leaf area components were quantified in the laboratory: any surface moisture was first wiped dry before placing a given leaf on a scanner (Epson V19, Epson, Tokyo, Japan) to digitize it. Then, Image J software (v1.51, USA) was used to measure its surface area (Shen et al., 2019). Next, each leaf was dried to a constant weight at 65°C and re‐weighed, and its SLA was calculated.

For each plot's leaf sample, a fully automatic Kjeldahl nitrogen analyzer (BUCHI, K‐360, Switzerland) was used to measure its leaf nitrogen content on a mass basis (LN, mg/g). A continuous flow rate analyzer (Skalar 1100, Skalar Analytical B.V., Delft, The Netherlands) was used to measure its leaf phosphorus content, likewise on a mass basis (LP, mg/g). A subset of leaf samples was combusted at high temperature in a FLASH 2000 elemental analyzer (PerkinElmer Inc., Waltham, MA, USA) to generate CO_2_. A Delta V Advantage stable gas isotope mass spectrometer (Thermo Fisher Scientific, Waltham, MA, USA) calculated the δ^13^C value of each sample by detecting the ratio of ^13^C to ^12^C of the emitted CO_2_, then comparing that value with international standard material (Pee Dee Belemnite [PDB]). To do that, the following formula was used:

$$\delta^{13}C=\left(R_{\mathrm{sa}}/R_{\mathrm{st}}-1\right)\times 1000$$

where δ^13^C denotes the carbon stable isotope data of the sample, whose value is in parts per thousand (‰); *R*_sa_ and *R*_st_ are the ^13^C/^12^C values of a given sample and international standards, respectively (Prave, 2000). The accuracy of δ^13^C was within 0.1‰.

#### 2.4.2 Belowground *Stipa* traits

Deionized water was used to clean the soil and remove impurities from the root surface. The entire root system of each plant was imaged with a scanner (Epson V19, Epson, Tokyo, Japan), and these images were then analyzed using WinRHIZO software (Version 6.0, OpusSoft, QC, Canada). Then, we quantified the total root length (TRL) and root tip number (RN). Next, the root samples were dried at 65°C to a constant weight before re‐weighing them. Root tissue density (RTD) was calculated as the ratio of root dry weight to root volume. To determine the root N and P contents (respectively, RN and RP), the same methodology was used as described above for the *Stipa* leaves.

### 2.5 Soil physical and chemical properties

Soil pH was measured by pH meter (PHS‐25, INESA Instrument, Shanghai, China) in a slurry (water:soil [v/m] = 2.5:1). Upon their arrival in the laboratory, all collected soil samples were immediately air‐dried, after which we removed any residual capillary roots and rocks. After grinding and passing the soil through a 0.15‐mm sieve, its total nitrogen (TN) and total phosphorus (TP) were determined in the same way as for the *Stipa* leaf and root samples. Soil organic matter content (SOC) was also quantified for each sample using the potassium dichromate external heating method. The volume of soil inside the ring cutter was measured and equated to the volume of the ring cutter itself, which had been placed in a Ziplock bag and labeled during the field survey work. Soil bulk density (BD, g/cm^3^) was measured using the cutting‐ring method (Deng et al., 2014).

### 2.6 Statistical analyses

We used MS Excel 2019 software (Microsoft Corporation, Redmond, WA, USA) for preliminary data sorting and processing. Redundancy analysis (RDA) was used to explore the relationship between functional traits and substitution distribution. Principal component analysis (PCA) was performed for the leaf traits (5 traits) and likewise for the root traits (6 traits), and again for the whole‐plant set of traits (11 traits) in Origin (Version 2019b, OriginLab Corporation, USA) to quantify and test the LES, RES, and WESP, respectively. Univariate analysis of variance (ANOVA) and multiple comparisons (LSD) were used to test for differences among economic spectra. Next, using the scores of the first two principal components (PC1 and PC2), standardized major axis (SMA) regressions were fitted (Warton et al., 2010), with their linear slopes and confidence intervals calculated. The R software package “smatr” (Venables et al., 2019) was used to determine the relationships between the three kinds of economic spectrums. Finally, to quantify and rank the effects of ecological factors on the aboveground traits, belowground traits, and whole‐plant trait set of *Stipa* species, variance decomposition (VD) and hierarchical partitioning (HP) were applied to the data, this implemented using the “rdacca.hp” package (Lai et al., 2022) in the R version 4.0.2 (R Core Team).

## 3 RESULTS

### 3.1 Functional traits of *Stipa* in Ningxia

The coefficient of variation (CV) values for aboveground functional traits across six *Stipa* species ranged ca. 15‐fold, from 3.31% to 43.65%, being least for δ^13^C and greatest for LP (Table 1). The average δ^13^C of *S. tianschanica* was the largest whereas that of *S. bungeana* was the smallest. For the LP, it was significantly highest in *S. breviflora* and lowest in *S. bungeana* (Table 1).

**TABLE 1:** Leaf functional traits of six species of *Stipa* in Ningxia (mean ± SD, *n* = 17).

Species	SLA	LN	LP	L(N/P)	δ^13^C
*S. glareosa*	65.23 ± 3.49a	32.55 ± 3.00a	2.75 ± 0.12ab	11.83 ± 0.70a	−27.36 ± 0.11b
*S. bungeana*	48.03 ± 2.75d	21.64 ± 1.30b	1.82 ± 0.26b	12.11 ± 2.31a	−27.37 ± 0.06b
*S. breviflora*	59.18 ± 4.98b	18.53 ± 5.009b	3.99 ± 1.43a	5.08 ± 1.97b	−26.00 ± 0.94a
*S. tianschanica*	53.82 ± 2.76c	13.32 ± 2.24bc	2.08 ± 0.21ab	5.61 ± 2.19b	−25.91 ± 0.16a
*S. grandis*	42.51 ± 4.12e	11.00 ± 0.87c	2.14 ± 0.33b	5.22 ± 0.81b	−26.42 ± 0.34a
*S. przewalskyi*	50.16 ± 1.71 cd	10.95 ± 0.84c	2.96 ± 0.13ab	3.7 ± 0.21b	−26.27 ± 0.17a
CV (%)	15.22	38.80	43.65	18.42	3.31

*Note*: CV, coefficient of variation (=SD/mean), expressed as a percentage. Different lowercase letters within the same column indicate significant differences (*p* < .05).

Trait abbreviations: L(N/P), the ratio of LN to LP; LN, leaf nitrogen content per mass; LP, leaf phosphorus content per mass; SLA, specific leaf area; δ^13^C, leaf carbon stable carbon isotope abundance value.

For the belowground functional traits of *Stipa*, their CV values ranged at least threefold across the six species, from 19.31% to 76.63%. Evidently, variation in RTN was the greatest, while the RN content was the lowest. The average RTN of *S. grandis* was the largest whereas that of *S. bungeana* was the smallest. For the RN, it was significantly highest in *S. glareosa* and lowest in *S. grandis* (Table 2).

**TABLE 2:** Root functional traits of six species of *Stipa* in Ningxia (mean ± SD, *n* = 17).

Species	RTD	RTN	TRL	RN	RP	R(N/P)
*S. glareosa*	0.74 ± 0.08b	14,597 ± 1790b	6510 ± 1099b	9.60 ± 0.50a	0.29 ± 0.06b	34.53 ± 7.52b
*S. bungeana*	0.68 ± 0.15b	2721 ± 808d	2006 ± 571d	7.84 ± 0.06b	0.19 ± 0.09b	48.53 ± 19.13a
*S. breviflora*	0.90 ± 0.34b	5783 ± 3146 cd	3051 ± 1436 cd	6.82 ± 1.15bc	0.40 ± 0.09a	18.81 ± 8.22c
*S. tianschanica*	0.54 ± 0.26b	5550 ± 2971 cd	2904 ± 1407 cd	7.20 ± 1.09bc	0.29 ± 0.04b	25.37 ± 3.59bc
*S. grandis*	0.86 ± 0.37b	11,054 ± 5806bc	5168 ± 2564bc	5.72 ± 0.90c	0.40 ± 0.05a	14.35 ± 2.97c
*S. przewalskyi*	2.33 ± 0.68a	24,739 ± 7084a	9480 ± 2620a	6.59 ± 1.08bc	0.28 ± 0.02b	23.10 ± 2.56c
CV (%)	56.22	76.63	61.64	19.31	27.41	51.09

*Note:* CV, coefficient of variation (=SD/mean), expressed as a percentage. Different lowercase letters within the same column indicate significant differences (*p* < .05).

Trait abbreviations: R(N/P), the ratio of RN to RP; RN, root nitrogen content per mass; RP, root phosphorus content per mass; RTD, root tissue density; RTN, root tip number; TRL, total root length.

### 3.2 Functional traits can explain the geographical substitution distribution of *Stipa*

**FIGURE 2:**
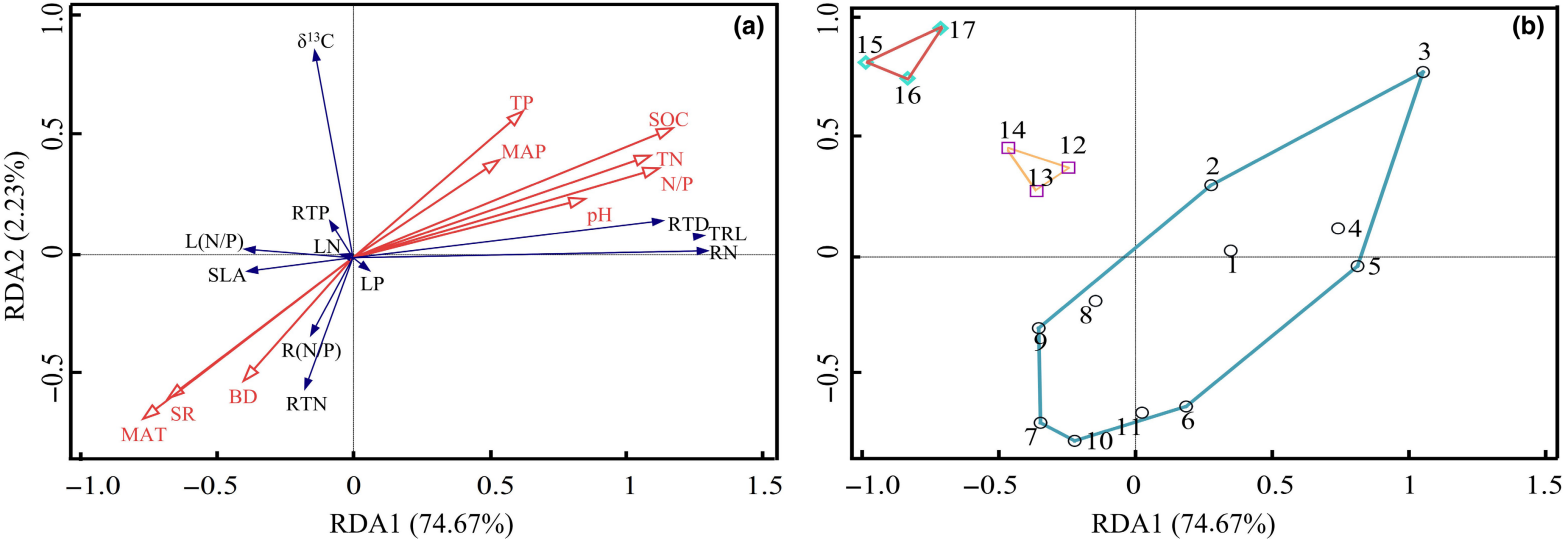
Redundancy analysis of environmental factors (in red in panel a) and functional traits (in black in panel a) of *Stipa* species. The numbers in panel (b) refer to the 17 plots sampled across Ningxia, China, which neatly clustered into three groups corresponding to desert (1–11), typical (12–14), and meadow (15–17) steppe types.

The RDA for the 11 leaf and root functional traits vis‐à‐vis the nine soil and climatic factors yielded a very high eigenvalue for axis 1 that was nearly 20 times that of axis 2, together accounting for 86.90% of the variation in the functional traits of *Stipa* (Figure 2a). Among the leaf functional traits, LN, L(N/P), LP, and SLA were positively correlated with MAT, SR, and BD, yet negatively correlated with other ecological factors. For δ^13^C, however, its correlation with the factors was almost orthogonal to that found for other five leaf functional traits. Among root functional traits, RTD, RN, RTP, and TRL were negatively correlated with MAT, SR, and BD, but positively correlated with the other factors. The correlation between R(N/P) and RTN vis‐à‐vis the ecological factors is exactly the opposite of the other four root functional traits. Meanwhile, we uncovered the pronounced aggregation of *Stipa* species according to the three types of grassland in the study area: desert steppe (plots 1–11), typical steppe (plots 12–14), and meadow steppe (plots 15–17) (Figure 2b). Altogether, these RDA results provided compelling evidence linking plant functional traits to the geographical distribution of species in this dominant genus.

### 3.3 Different spectra and their relation in types of grassland

**FIGURE 3:**
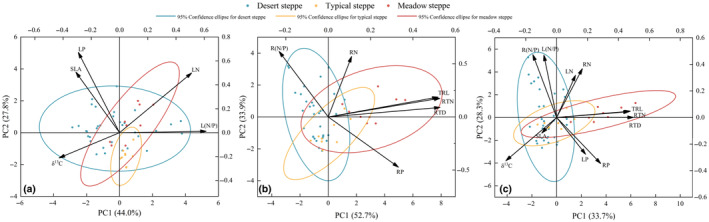
Principal components analysis (PCA) of the (a) leaf traits, (b) root traits, and (c) whole‐plant traits of *Stipa* species in the three grassland habitat types across Ningxia, China.

The PCA results (Figure 3) could be used to discern the resource utilization strategies of different *Stipa* species in the three grassland habitat types. Specifically, where the contribution (explanatory strength) of traits is relatively large along the first (PC1) or second (PC2) axis (Table 3), this could indicate whether the LES, RES, or WPES applied to *Stipa* species in different grassland habitats. The loadings of *S. glareosa*, *S. bungeana*, *S. breviflora*, and *S. tianschanica* distributed in desert steppe were mainly situated in the positive semi‐axis region of PC1 and PC2, indicative of a “quick investment‐return” acquisition strategy. However, the loadings of species dominating the typical steppe (*S. grandis*) and meadow steppe (*S. przewalskyi*) were mainly positioned in the negative semi‐axis region of PC1 and PC2, which suggested that *Stipa* growing in these two grassland types followed a “slow investment‐return” conservative strategy. The PCA revealed show that PC1 and PC2 together explained 71.2% of the variance in leaf traits (Figure 3a), 86.8% of the variance in root traits (Figure 3b), and 61.9% of the variance in whole‐plant traits (Figure 3c). In summary, at both the root and whole‐plant levels, *Stipa* has apparently adopted the same investment approach in various grassland types. In both desert and typical steppe, the “quick investment‐return” strategy was prevalent, whereas all *Stipa* spp. relied on a “slow investment‐return” strategy in the meadow steppe habitat.

**TABLE 3:** Differing PCA scores among the desert, typical, and meadow steppes.

Economic spectrum	Grassland type	Mean ± SE
Leaf	Desert steppe	3.98 ± 1.43 B
	Typical steppe	3.45 ± 1.04 A
	Meadow steppe	4.58 ± 0.97 A
Root	Desert steppe	3.64 + 1.36 B
	Typical steppe	3.50 ± 1.02 B
	Meadow steppe	5.77 ± 1.74 A
Whole‐plant	Desert steppe	3.59 ± 1.69 B
	Typical steppe	3.65 ± 0.77 B
	Meadow steppe	5.83 ± 2.04 A

*Note*: Different capital letters indicate significant differences (*p* < .05) within each spectrum.

**FIGURE 4:**
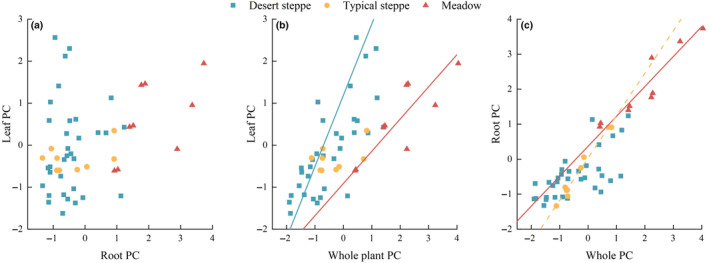
Standard major axis (SMA) regressions among the leaf PC, root PC, and whole‐plant PC of *Stipa* in the three grassland habitat types across Ningxia, China. Linear relationships of (a) root PC1 and leaf PC, (b) leaf PC1 and whole‐plant PC, and (c) root PC and whole‐plant PC. Only the fitted lines of significant regressions are shown (*p* < .05), with isometric growth plotted in as a solid line and allometric growth as a dashed line. PC (A single coordinate) = ((contribution rate of PC1/(contribution rate of PC1 + contribution rate of PC2))*loading value on PC1) + ((contribution rate of PC2/(contribution rate of PC1 + contribution rate of PC2))*loading value on PC2).

According to the SMA regressions, irrespective of the grassland habitat, there was no significant allometric relationship between the PCs of leaf and root traits (all three *p*‐values > .05) (Figure 4a). However, there was a significant isometric growth (*p* < .05) between leaf PC and the whole‐plant PC in both desert steppe (95% confidence interval, CI = 0.91–1.48) and meadow steppes (CI = 0.47–1.22). In contrast, root PC displayed a significant allometric growth relationship with the whole‐plant PC in typical steppe (CI = 1.06–1.38), while an isometric growth was found between root PC and overall plant PC in meadow steppe (CI = 0.63–1.15).

### 3.4 Main ecological factors driving species substitution distribution of *Stipa* species

On a larger spatial scale, abiotic ecological factors may exert hydrothermal effects on the growth environment of *Stipa* species. Accordingly, we also explored the variance decomposition of soil and climate factors potentially influencing the functional traits of *Stipa* plants across Ningxia. Soil physical and chemical properties and climate factors together accounted for 94.7% of the variance in aboveground functional traits of *Stipa*, leaving just 5.3% of it unexplained. Among the factors, the explanatory strengths of soil and climatic factors were, respectively, 31.5% and 17.6%, whose co‐explanatory contribution was 45.6%. Remarkably, soil factors and climatic factors together explained nearly all (97.1%) of the variance in *Stipa*'s belowground functional traits, with only 2.9% left unexplained. The explanatory strength of soil factors (41.3%) was at least double that of climatic factors (19.5%) and also higher than their co‐explanatory strength (35.1%).

**FIGURE 5:**
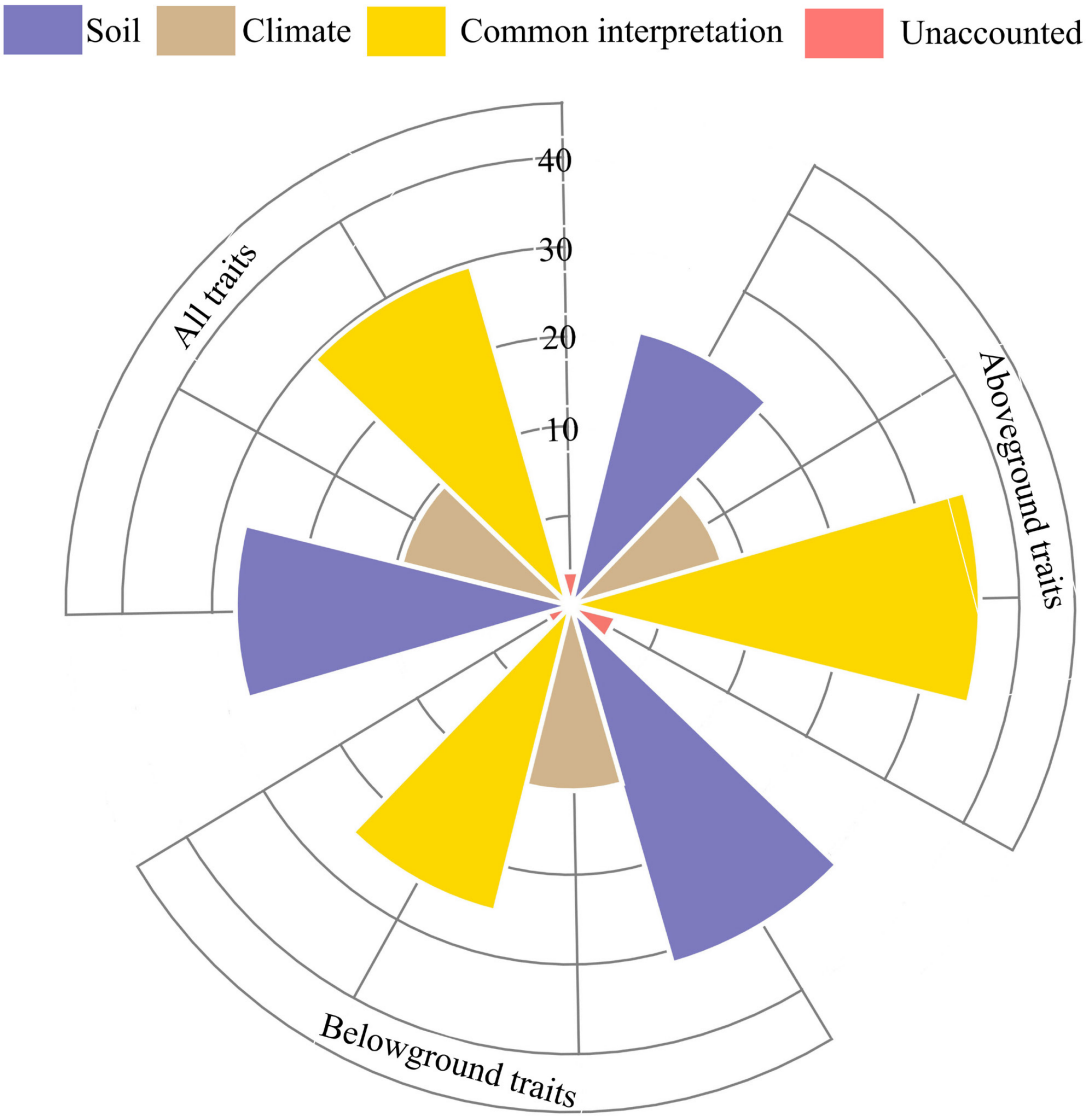
Rosette diagram showing the variance decomposition of functional traits of *Stipa* according to different levels of the soil and climate factor examined.

Concerning the whole‐plant trait set for *Stipa*, only 3.9% of its variance went unexplained, with 96.1% of it explained by all ecological factors in concert: 37.4% by soil factors, 19.5% by climatic factors, and 39.2% from their co‐explanatory contribution. Hence, across differing trait variance decomposition levels, the explanatory strength of soil factors always surpassed that of climatic factors (Figure 5).

**FIGURE 6:**
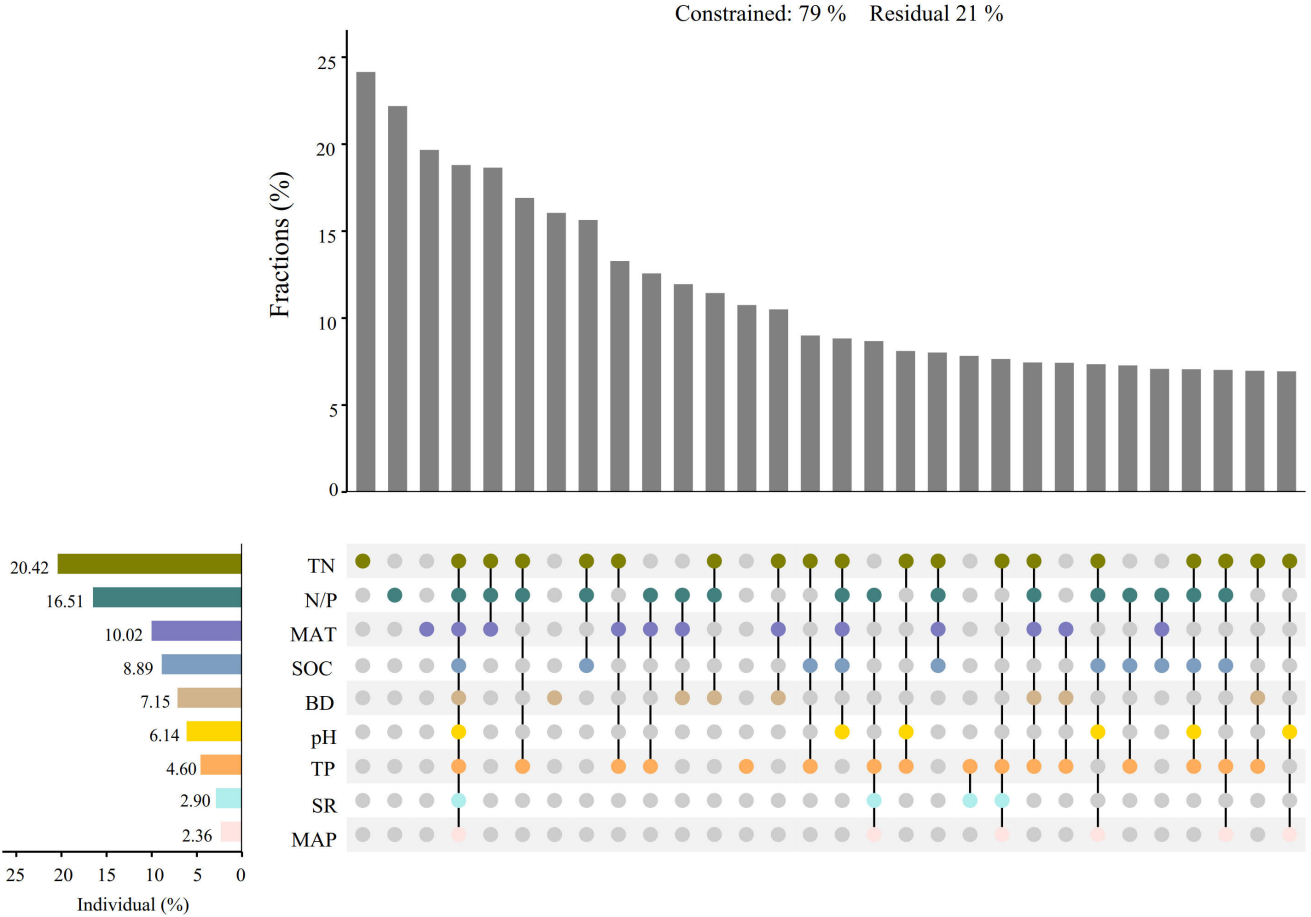
Effects of ecological factors on *Stipa*. Trait abbreviations are TN (soil total nitrogen), TP (soil total phosphorus), N/P (the ratio of TN to TP), MAT (mean annual temperature), MAP (mean annual precipitation), BD (soil bulk density), SOC (soil organic carbon content), and SR (amount of solar radiation). At bottom‐left, numbers are the percentage variance explained by each environmental factor. The dot matrix and vertical bars indicate values associated with shared and exclusive contributions from each factor.

To further explore the relation between ecological factors and the distribution of *Stipa* species, the hierarchical segmentation method was used to screen and rank the nine ecological impact factors (Figure 6). Their ranking was as follows: TN > N/P > MAT > SOC > BD > pH > TP > SR > MAP.

## 4 DISCUSSION

### 4.1 Basic characteristics of plant functional traits in *Stipa*

*Stipa*, as the dominant genus in China's natural grassland, is also the prevailing plant taxon in the Eurasian Grassland (Yu et al., 2015). Given that most *Stipa* species are integral components of these grasslands, especially in their successional dynamics, these plants play a prominent role in maintaining the general stability of grassland community structure on a continental scale (Ye, Liu, Chang, Shan, & Fu, 2020; Ye, Liu, Chang, Shan, Mu, et al., 2020). The coefficient of variation (CV) of one or more leaf functional traits can be used to gauge leaf adaptability to environmental changes, with a higher CV indicating a greater plasticity for environmental adaptation (Valladares et al., 2000). Prior research has demonstrated that, for *Stipa* spp., their intra‐species variation in functional traits is generally below 30% (Zhao et al., 2006), in stark contrast to our result. Here, we find a larger CV for the belowground than aboveground functional traits of six *Stipa* species. This greater variability in root traits could be explained by the more complex and heterogeneous habitat of roots, which led to higher variability in their phenotypic trait expression. Overall, the leaf N/P ratio was less than 14 for *Stipa*, indicating that nitrogen restricted the growth of species in this genus, whereas its root N/P ratio exceeded 16 (excluding *S. grandis*), indicating that phosphorus limitation is a pertinent factor in their growth. Differences in limiting elements reflect the allocation of resources, both material and energy, between the aboveground and belowground components of plants, thereby ensuring a more stable growth trajectory (Raven, 2022).

### 4.2 Functional trait variability in *Stipa* species: insights into substitution distribution

By examining the niche differentiation and substitution distribution of *Stipa* species in Ningxia's natural grasslands, we can delve deeper into how these species could have adapted to different ecological environments via niche differentiation, which in turn shapes their spatial pattern of substitution distribution. Functional traits are the foundation of plant adaptation to the environment and niche differentiation (Wang et al., 2022). This sensitivity and adaptability to environmental factors are conducive to the niche differentiation of *Stipa* species. In particular, *S. breviflora* could have a niche that is more closely related to water utilization efficiency and drought resistance in desert steppe (Ye, Liu, Chang, Shan, & Fu, 2020; Ye, Liu, Chang, Shan, Mu, et al., 2020), while *S. bungeana* may be better adapted for seed dispersal and attaining wider distribution (Hu et al., 2014). The survival strategies of *S. tianschanica* under extreme drought conditions likely enable it to occupy a unique niche in the desert steppe habitat (Yin et al., 2022). In typical steppe, *S. grandis* may dominate there due to its higher biomass and productivity. The niche of *S. przewalskii* in meadow steppe might be associated with its growth rate and reproductive capacity under ample water conditions.

Substitution distribution is the external integration of functional traits and niche attributes (Guisan et al., 2019). Since plants can adjust their functional traits to adapt to their immediate environment, they could come to occupy a specific niche within the local community (Chen et al., 2022). This niche differentiation process results in the spatial substitution distribution of different species, thereby jointly maintaining the stability and diversity of a grassland ecosystem. Our study revealed the patterns of substitution distribution of *Stipa* species in natural grasslands of Ningxia, further illustrating the relationships among functional traits, niche attributes, and substitution distribution. These research findings can inform the ecological restoration and reconstruction of grassland systems. By understanding and utilizing the functional traits and niche attributes of plants, appropriate species could be chosen for vegetation restoration, thereby enhancing the stability and resilience of the ecosystem to disturbances (Carlucci et al., 2020).

### 4.3 Different economic spectra of *Stipa* and their relationship

To maintain their own growth and development, plants need to effectively use and distribute the environmental resources they acquire, to achieve “optimization” (Wright et al., 2005). According to our study's results, the *Stipa* in desert steppe are all situated on the “quick investment‐return” end of the LES (leaf economic spectrum), RES (root economic spectrum), and WPES (whole‐plant economic spectrum). This may be a strategy to quickly gain a growth advantage under harsh environmental conditions. However, the research findings are opposite to those of Huo et al. (2022) for the desert in the Qaidam Basin. This discrepancy may be due to the scarce water and nutrient resources in that Basin, which require plants to use these resources more efficiently, thus adopting a slow‐return investment strategy. Although the *Stipa* in typical steppe is also characterized by a “quick investment‐return” on both the LES and WPES, it lies at the “slow investment‐return” end of the RES. This could be because investments in LES yield higher returns for the growth of large *Stipa* species. However, in terms of RES and WPES, typical steppe's *Stipa* still adopted a rapid investment strategy, indicating a potentially higher demand for water and nutrients. This indicates that, even within the same species, strategies across different economic spectra can vary based on physiological traits and environmental demands (Wang et al., 2021). The *S. przewalskyi* in meadow steppe always conforms to the “slow investment‐return” end, whether on the LES, RES, or WPES. This could be because the environmental conditions in meadow steppes are relatively favorable, allowing plants to more easily access water and nutrients, thus obviating the need for rapid investments to compete for growth advantages (Carvajal et al., 2019).

In desert grasslands, we observed an isometric relationship between the LES and WPES, suggesting that *Stipa* spp. adapt to water‐limiting and nutrient‐poor conditions by maintaining synchronous growth in leaves and at the whole‐plant level, and by investing in leaf nitrogen to enhance drought tolerance and nutrient acquisition. This is analogous to Wink's (2013) findings on legume plants, whose the production and accumulation of nitrogen‐rich osmolytes contribute to internal osmotic adjustments, which lowers their water potential and facilitates water uptake from dry soil, bolstering the drought resistance of these plants, in a way similar to our study results. In contrast, within typical grasslands, we found an allometric growth relationship (slope > 1) between RES and WPES. *Stipa* may increase its investment in root systems to ensure that its water and nutrient uptake capabilities are matched with its photosynthetic output, thereby maintaining growth and productivity. Therefore, plants might enhance root investments to explore deeper soil layers or broader areas to access more abundant water and nutrient resources (Wen et al., 2021). In meadow grasslands, both the LES and RES exhibited isometric relationships with the WPES. This balanced strategy should help *Stipa* plants maintain stable growth and productivity in resource‐rich environments while avoiding wasteful resource allocation (Zan et al., 2024). It is noteworthy that no significant allometric or isometric relationship emerged between RES and LES, which may reflect the *Stipa*'s flexibility in resource allocation. This flexibility likely allows plants to adjust their investment proportions based on current environmental conditions and growth stages, enabling them to adapt to various grassland types pressures and optimize resource utilization, thereby enhancing their survival and growth capabilities. Wang et al. (2021) reported a similar decoupling phenomenon in deciduous forests, where LES and RES did not always change in synchrony, suggesting that leaves and roots face different environmental constraints and functional demands.

### 4.4 Dominant factors affecting the substitution distribution of *Stipa* species

Plant distributions can take multiple forms, but generally they are shaped by two distinct categories of ecological factors: abiotic and biotic. Abiotic factors encompass climate and soil, both of which can substantially influence how plant species are distributed in space and time. In contrast to the study by Feng et al. (2010) that examined the ecological factors affecting the functional traits of dominant oak tree species in the temperate zone along a north–south transect in eastern China, our findings indicate that soil factors play a more significant role than climatic factors in influencing the aboveground, belowground, and whole‐plant functional traits of *Stipa* grass species. In the natural grasslands of Ningxia, the variation in climatic factors between its north and south can range from 3.35% to 22.57%, while that of soil ranges from 13.71% to 82.01%. Clearly, there is inherently more local variation in soil than climate, which could explain why soil factors explain more of the plant functional traits of *Stipa* than do climatic factors.

Our study also employed a hierarchical partitioning method to screen and rank nine ecological impact factors (Figure 6), revealing that soil factors played a pivotal role in the substitutional distribution of *Stipa* species. Nitrogen and phosphorus are essential elements for plant growth, being crucial throughout this growth process. Hence, their respective content and their ratio in the soil likely influence *Stipa*'s plant development. In desert steppe, due to the scarcity of nitrogen, *Stipa* species would be expected to follow a rapid investment strategy. There they could enhance photosynthetic efficiency through the development of a robust root system and an increased leaf area to adapt to drought and poor soil conditions (Yang & Luo, 2011). We found that the impact of annual mean temperature (MAT) exceeded that of annual precipitation (MAP), which might have several reasons. The sensitivity of *Stipa* species to temperature changes could be tied to the characteristics of arid and semi‐arid regions. Their temperature may directly affect plant photosynthesis, metabolic processes, and the nutrient cycling of ecosystems, thus influencing *Stipa* plants' growth. In comparison, water conditions there are relatively stable, making temperature a more influential factor. In typical steppe, *Stipa* species still employed a rapid investment strategy, indicating their higher demand for water and nutrients. This result suggests that environmental resources may limit their growth more. It also indicates that, even when water is relatively abundant, other abiotic factors such as temperature might still play a critical role in *Stipa*'s growth.

In a previous study, Yang (2016) combined a maximum extraction model with remote sensing data to obtain the suitable distribution area of *Stipa* plants, which is consistent with the species distributions in the present paper. That is, *S. breviflora*, *S. glareosa*, and *S. bungeana* are all distributed in desert grassland, while *S. grandis* is distributed in the typical steppe habitat where water conditions are better for its growth. Shi et al. (2014) explored the ecological niche of *Stipa* species that had been fenced for varying durations (up to 30 years) and discovered that *S. przewalskyi* replaced *S. grandis* as the fenced years increased, similar to our finding that *S. przewalskyi* is distributed in the meadow steppe which provides it with a better habitat. As our results show, soil factors strongly affect the functional traits of the six *Stipa* species, leading to their coupling and thus geographical substitution distribution among different types of natural grasslands. We therefore postulate that the alternative distribution of *Stipa* is the outcome of trait adaptation to soil patterning, especially soil total nitrogen and soil total phosphorus. Management practices, whether traditional or modern, could likely influence the distribution and functional traits of *Stipa* species by altering soil properties as well as water availability. Traditional rotational grazing (Byrnes et al., 2018) and crop rotation (Yang et al., 2023) can modify nutrient cycling and community structure, while modern land improvement and water management strategies can directly impact soil texture and water accessibility (Blanco & Lal, 2023), which are crucial factors for *Stipa* growth (Wen et al., 2022). These management measures may influence the distribution of *Stipa* in various grassland types and their niche differentiation. Therefore, understanding the relationship between management interventions, soil patterns, and *Stipa* functional traits is vital for predicting and managing future grassland changes. By combining management strategies with soil patterns and the adaptive functional traits of *Stipa*, we can better comprehend how *Stipa* communities maintain an ecological balance in natural grasslands and how management interventions can promote the health and sustainability of grassland ecosystems.

## 5 CONCLUSION

This study examined functional trait variability among six species of *Stipa* in the natural grasslands in Ningxia and its correlation with their substitution distribution. The results indicate significant disparities in functional traits among *Stipa* species, which are closely linked to local soil and climatic factors. These trait differences are likely instrumental in explaining the species' distribution across diverse grassland types. *Stipa* species exhibit distinct strategies for resource utilization according to grassland type, this is accompanied by an economic spectrum of coordination between leaves, roots, and the overall plant. That coordination and its associated trade‐offs can enrich our understanding of the adaptive strategies of *Stipa* species under various environmental conditions, which could play a key role in explaining and predicting their geographical patterns of substitution distribution. Nonetheless, this study's constraints lie in the limited array of functional traits assessed and the absence of long‐term data, hindering a full evaluation of *Stipa*'s functional diversity and trait dynamics over time. Future work should broaden the scope of measured traits, initiate long‐term monitoring, and utilize modeling to forecast trait environment interactions. Furthermore, investigating divergent patterns in functional traits among plant functional groups and their distributional patterns, as well as the genetic underpinnings and evolutionary drivers of *Stipa*'s traits, is crucial. The knowledge gained will enhance our grasp of how plant functional traits shape their geographical dispersal and responses to a changing environment.

## AUTHOR CONTRIBUTIONS

**Jun Yang:** Conceptualization (equal); methodology (equal); visualization (lead); writing – original draft (lead); writing – review and editing (lead). **Xiaowei Li:** Data curation (lead); funding acquisition (lead); project administration (lead); resources (lead); validation (lead). **Junlong Yang:** Conceptualization (equal); investigation (equal); resources (equal); supervision (equal). **Shuang Yu:** Data curation (equal); formal analysis (equal); software (equal); supervision (equal). **Hongmei Zhang:** Project administration (equal); supervision (equal). **Bo Yang:** Conceptualization (equal); supervision (equal); validation (equal).

## Data Availability

The data that support the findings of this study are openly available in https://doi.org/10.6084/m9.figshare.25111853.v1.
